# Dual bloom of green algae and purple bacteria in an extremely shallow soda pan

**DOI:** 10.1007/s00792-019-01098-4

**Published:** 2019-05-13

**Authors:** Kristóf Korponai, Attila Szabó, Boglárka Somogyi, Emil Boros, Andrea K. Borsodi, Laura Jurecska, Lajos Vörös, Tamás Felföldi

**Affiliations:** 1 Department of Microbiology, ELTE Eötvös Loránd University, Pázmány Péter stny. 1/c., Budapest, 1117 Hungary; 2 Balaton Limnological Institute, MTA Centre for Ecological Research, Klebelsberg Kuno u. 3., Tihany, 8237 Hungary

**Keywords:** Soda pan, Purple bacteria, *Oocystis*, *Thiorhodospira*, *Rhodobaca*, Bloom

## Abstract

In April 2014, dual bloom of green algae and purple bacteria occurred in a shallow, alkaline soda pan (Kiskunság National Park, Hungary). The water was only 5 cm deep, in which an upper green layer was clearly separated from a near-sediment purple one. Based on microscopy and DNA-based identification, the upper was inhabited by a dense population of the planktonic green alga, *Oocystis submarina* Lagerheim, while the deeper layer was formed by purple, bacteriochlorophyll-containing bacteria, predominated by *Thiorhodospira* and *Rhodobaca*. Additional bacterial taxa with a presumed capability of anoxygenic phototrophic growth belonged to the genera *Loktanella* and *Porphyrobacter*. Comparing the bacterial community of the purple layer with a former blooming event in a nearby soda pan, similar functional but different taxonomic composition was revealed. Members from many dominant bacterial groups were successfully cultivated including potentially new species, which could be the result of the application of newly designed media.

**Electronic supplementary material:**

The online version of this article (10.1007/s00792-019-01098-4) contains supplementary material, which is available to authorized users.

## Introduction

Soda lakes have Na^+^- and CO_3_^2−^/HCO_3_^−^-dominated alkaline water, and therefore, they are different from other athalassohaline waters (Boros et al. 2014; Boros and Kolpakova 2018). Soda lakes can be found in almost every continent (Grant 2006; Sorokin et al. 2014; Boros and Kolpakova 2018): in Central Asia (Kulunda Steppe), Inner Asia, East Africa (Eastern Rift Valley), Central Europe (Carpathian Basin) and sporadically in India (Lonar Lake) and North America (Mono Lake and Soap Lake). These alkaline and saline environments range from deep meromictic to shallow lakes, and can be grouped into hypersaline (> 50 g/L) and less saline water bodies.

Shallow lakes and pans are characteristic features of the semiarid steppe and undergo significant diurnal (mainly temperature and oxygen concentration) and annual (mainly temperature, volume and salinity) changes regarding various physical parameters. According to our current knowledge, within Europe, soda lakes could be found exclusively in the Carpathian Basin; their size ranges from small wetlands of few m^2^ to few hundred ha (Boros et al. 2014, 2017). Most of them are intermittent aquatic systems and have low water transparency (Boros et al. 2017; Somogyi et al. 2017). Two main types of these shallow lakes (which are usually referred as pans) could be distinguished, the ‘turbid type’ (high amount of suspended solids and usually high concentration of humic substances) and the ‘colored type’ (relatively low amount of suspended solids and very high concentration of humic substances) (Boros et al. 2017). The ‘fluid sediment’ concept has been proposed for the turbid water type, as wind induces continuous sediment resuspension due to their extreme shallowness (< 50 cm) (Eiler et al. 2003; Boros et al. 2017).

The key primary producers in soda lakes of the Carpathian Basin are cyanobacteria, eukaryotic green algae and euglenophytes (Vörös et al. 2008; Somogyi et al. 2017). Phytobenthos is considered to be negligible due to the strong underwater light limitation (Boros et al. 2013), while phytoplankton (especially in the turbid-type waters) is usually dominated by pico-sized (< 3 μm) species (Vörös et al. 2008; Felföldi et al. 2009; Somogyi et al. 2009, 2017) and has characteristic seasonal changes. Below 15 °C, picoeukaryotes (mainly *Chloroparva* and *Choricystis*) dominate, while above this temperature picocyanobacteria (mostly *Synechococcus/Cyanobium*) occur (Vörös et al. 2008; Felföldi et al. 2009, 2011; Somogyi et al. 2009, 2011, 2016). Interestingly, pico-sized green algae regularly bloom under the ice during winter (Somogyi et al. 2009; Pálffy et al. 2014); while in spring and summer, blooms of larger (mainly green) algae could be formed due to the high productivity of these waters, which occasionally co-occur with mass production of purple bacteria near the sediment surface (Borsodi et al. 2013).

The aim of the present study was the characterization of a dual bloom, which occurred in a soda pan with a special focus on the bacterial community inhabiting the purple layer. Comparing the obtained results (physicochemical parameters and taxonomic composition) with a similar, previous dual bloom event reported from the studied region (Borsodi et al. 2013), the underlying factors shaping the bacterial community composition (BCC) of these *Oocystis*-associated purple bacterial blooms were revealed.

## Materials and methods

### Study site and sample collection

An anonymous, small, turbid soda pan (N46°45.818′, E19°10.828′), located in the Kiskunság National Park near Soltszentimre (Hungary, Central Europe), was sampled on 23 April 2014. In their great cadastre, Boros et al. (2013) designated it as anonymous lake no. 60. Water depth reportedly fluctuates between 0 and  ~ 60 cm; the average water surface is 0.58 ha (Boros et al. 2013). Pan water is dominated by Na^+^ and HCO_3_^− ^>Cl^−^ (Table 1, Supplementary Figure 1), which is supplied mainly by groundwater and precipitation (Simon et al. 2011), and neither has vegetation cover nor surface inflow or outflow (Boros et al. 2013).

**Table 1:** Basic characteristics of the studied anonymous soda pan water and measured physicochemical parameters during the dual bloom Previous data (2009 and 2010) were taken from Boros et al. (2013)

Parameters	17 May 2009	6 March 2010	23 April 2014
Depth (cm)	5	58	5
pH	9.47	9.02	10.16
Conductivity (mS/cm)	33.0	4.8	15.5
O_2_ conc. (mg/L)	13.4	14.3	n.d.
Pt color (mg/L)	516	n.d.	n.d.
Turbidity (NTU)	n.d.	n.d.	490
TSS (mg/L)	1307	76.7	n.d.
K^+^ (mg/L)	7.6	n.d.	n.d.
Na^+^ (mg/L)	1145	n.d.	n.d.
Ca^2+^ (mg/L)	7.4	n.d.	n.d.
Mg^2+^ (mg/L)	9.3	n.d.	n.d.
SO_4_^2−^ (mg/L)	255	n.d.	370
Cl^−^ (mg/L)	592	n.d.	n.d.
HCO_3_^−^ (mg/L)	1893	n.d.	n.d.
CO_3_^2−^ (mg/L)	47.0	n.d.	n.d.
NH_4_^+^-N (mg/L)	n.d.	n.d.	19.8
NO_2_^−^-N (mg/L)	n.d.	n.d.	< 0.01
NO_3_^−^-N (mg/L)	n.d.	n.d.	47.0
TN (mg(L)	n.d.	n.d.	135
SRP (mg/L)	2.6	n.d.	37
TOC (mg/L)	n.d.	n.d.	1143
Chl *a* (mg/L)	1.0	n.d.	10.6^a^

*NTU* nephelometric turbidity unit, *TN* total nitrogen, *TOC* total organic carbon, *SRP* soluble reactive phosphorous, *n.d.* not determined

^a^Data represent only the upper, green layer

A cylinder was used for sample collection, and subsamples were taken from the upper, green-colored water and from the purple layer over the sediment using a pipette. Determination of the BCC was carried out only from the latter.

### Limnological and water chemistry analyses

Basic physicochemical parameters were determined on site using a MultiLine P 8211 multimeter (WTW). Chemical analyses were performed under laboratory conditions according to the methods described in detail in Felföldi et al. (2016).

For pigment analyses, the samples were filtered through a GF-5 glass fiber filter (Whatman). Chlorophyll *a* (Chl *a*) and bacteriochlorophyll *a* (Bchl *a*) were extracted from the filters as described in Felföldi et al. (2016), while another filter set per se was used for in vivo absorption measurements. Chlorophyll concentration was measured using the method of Wellburn (1994), and the concentration of Bchl *a* was determined according to Biel (1986). In vivo absorption spectra were recorded with a 160A UV–Vis spectrophotometer (Shimadzu) between 380 and 900 nm.

Native preparations from the two layers were photographed using an Olympus BX51 microscope with a CCD camera (Olympus DP71).

### Determination of BCC based on total DNA analysis

Community DNA was extracted according to Szabó et al. (2017). For the determination of BCC, next-generation DNA sequencing (NGS) was applied using the protocol and sequence analysis pipeline as described previously (Szabó et al. 2017). For this, the V3–V4 region of the 16S rRNA gene was amplified with S-D-Bact-0341-b-S-17 forward (5′-CCTACGGGNGGCWGCAG-3′) and S-D-Bact-0785-a-A-21 reverse (5′-GACTACHVGGGTATCTAATCC-3′) primers (Klindworth et al. 2013). Based on the proposed species-level sequence similarity threshold (Tindall et al. 2010), operational taxonomic units (OTUs) were picked at 97% similarity. Raw sequence data are available under the NCBI BioSample ID SAMN10724752. Sequences of cultivated strains (see below), and those (strains and clones) from the purple bacterial bloom published in Borsodi et al. (2013) were also analyzed using the same pipeline. Since plastid 16S rRNA gene references are underrepresented in the ARB-SILVA database, chloroplast sequences were analyzed separately as described in detail by Kalwasińska et al. (2017).

The ratio of Archaea and Bacteria was determined with qPCR targeting the 16S rRNA gene. All reactions were carried out in triplicates in a StepOnePlus Real-Time PCR System (Thermo Fisher Scientific) using TaqMan Gene Expression Master Mix (Thermo Fisher Scientific). A final volume of 16 μL was applied with 1 × Taqman Gene Expression Master Mix and 1 μL DNA sample. In case of the Bacteria-specific reaction, the following primers and probe were applied: 1.2 μM BACT1369F (5′-CGGTGAATACGTTCYCGG-3′), 1.0 μM PROK1492R (5′-GGWTACCTTGTTACGACTT-3′), 0.5 μM TM1389F (5′-FAM-CTTGTACACACCGCCCGTC-BHQ-3′) (Suzuki et al. 2000); while for Archaea, 0.8 μM Arch349F (5′-GYGCASCAGKCGMGAAW-3′), 0.8 μM Arch806R (5′-GGACTACVSGGGTATCTAAT-3′) and 0.5 μM Arch516F (5′-FAM-TGYCAGCCGCCGCGGTAAHACCVGC-BHQ-3′) primers and specific probe were used (Takai and Horikoshi 2000). Reactions were carried out using the thermal profile: 50 °C for 2 min, 95 °C for 10 min, followed by 40 cycles at 95 °C for 15 s and 60 s at 56 °C for the Bacteria- and at 59 °C for the Archaea-specific reaction. Standard curves were used for the estimation of 16S rRNA gene numbers using the StepOne v2.3 software (Thermo Fisher Scientific) based on serial tenfold dilution (10^9^–10^3^ copy) of genomic DNA from *Nitrincola lacisaponensis* DSM 16316^T^ and *Thermoplasma acidopilum* DSM 1728^T^. Gene copy numbers were determined based on the molar mass values of the standard amplicons.

### Analyses based on bacterial strain cultivation

For cultivation of bacteria, three different media were used: ‘R’ [DSMZ medium 830 (R2A; see details: http://dsmz.de), pH 10, adjusted with 1 M NaOH], ‘C’ [1 L autoclaved sample water, 16 g gellan gum (Gelzan CM, Sigma), 0.6 g MgSO_4_ × 7H_2_O and 0.3 g CaCl_2_ × 2H_2_O)] and ‘S’ (1 L dH_2_O, 16 g gellan gum, 1 g yeast extract, 0.6 g MgSO_4_ × 7H_2_O, 0.3 g CaCl_2_ × 2H_2_O and 10 g NaHCO_3_; pH 10, adjusted with 1 M NaOH). A tenfold dilution series using sterile distilled water was prepared from the sample, and aliquots were subsequently spread onto solid media. Incubations were carried out under both aerobic and anaerobic (Microbiology Anaerocult^®^ A mini, Merck) conditions at room temperature under scattered light. Colony counts were recorded after 19 days, then randomly picked colonies were isolated and purified; the obtained strains were used for downstream analyses (the first letter of the stain codes is according to the applied media).

DNA was extracted from bacterial strains with the G-spin™ Total DNA Extraction Kit (iNtRON Biotechnology). The 16S rRNA gene was amplified by PCR using the primers 27F (Lane 1991) and 1492R (Polz and Cavanaugh 1998). PCR products were purified and sequenced by LGC Genomics (Berlin, Germany). Chromatograms were manually corrected with Chromas (Technelysium). For taxonomic identification, EzBioCloud’s online service was used (Yoon et al. 2017). Sequences were screened for chimeras using Pintail 1.0 (Ashelford et al. 2005). GenBank accession numbers of the obtained sequences are: KR233183–KR233245.

Measuring the Bchl concentration using the spectroscopy-based method of Biel (1986) was not possible due to the weak growth rate of strains which yielded low amount of biomass. Therefore, microscopy- and PCR-based techniques were applied to assess the phototrophic potential of the strains. Briefly, the cells were detected with an Olympus BX51 epifluorescence microscope upon excitation at 350–550 nm (excitation of Bchl *a*) using a 780-nm high-pass filter and a monochrome CCD (Olympus XM10) camera according to Jiao et al. (2006). All strains were checked for the presence of the *pufM* gene (coding the M subunit of the heterodimeric cores of the photosynthetic reaction center complex) with PCR using the primers pufM_uniFfresh and pufM_uniRfresh (Martinez-Garcia et al. 2012). The 25 µL reaction volume contained 1 × Taq buffer (Fermentas), 2 mM MgCl_2_, 0.2 mM dNTP, 0.325 μM of each primer, 1 U *Taq* polymerase (Fermentas), 10 μg BSA (Fermentas) and 1 µL DNA. Cycling conditions were as given by Martinez-Garcia et al. (2012). Amplicons were sequenced and processed as described above. GenBank accession numbers of the obtained sequences are: KX361312–KX361323.

## Results

### Results of limnological analyses

At the time of sampling, pH 10.2 and 15.5 mS/cm conductivity were measured (Table 1), which is equivalent to 12.4 g/L salinity according to the conversion coefficient determined for these pans by Boros et al. (2014). Other measured parameters of lake water are shown in Table 1. High levels of total organic carbon (TOC), nitrogen forms, orthophosphate and sulfate were present. The upper layer of the water contained 10.6 mg/L Chl *a* and 1.9 mg/L Bchl *a*, while the lower contained 4.0 mg/L Chl *a* and 7.8 mg/L Bchl *a*. *Oocystis submarina* Lagerheim, a unicellular green alga, was identified microscopically as the sole organism causing the algal bloom (Supplementary Figure S2). Based on microscopy, high density of vibrio-shaped bacteria was observed in the bottom layer. These bacteria showed phototaxis and sulfur globules were also observed (Supplementary Figure S3).

### Results of cultivation-based analyses

The three media used for the determination of heterotrophic bacterial viable cell counts in the purple layer resulted in different plate counts. Under aerobic conditions 1.7 × 10^6^, 2.8 × 10^7^ and 3.5 × 10^7^ CFU (colony forming units)/mL were recorded after 19 days of incubation on medium ‘C’, ‘R’ and ‘S’, respectively. Anaerobic cultivation yielded lower or similar values: 4.2 × 10^4^ and 3.6 × 10^7^ CFU/mL were obtained after 19 days in the case of media ‘C’ and ‘S’, respectively, while no colony formation was observed using medium ‘R’. Therefore, medium ‘S’ was the most suitable for the growth of facultative anaerobic bacteria present in the sample.

**Fig. 1:**
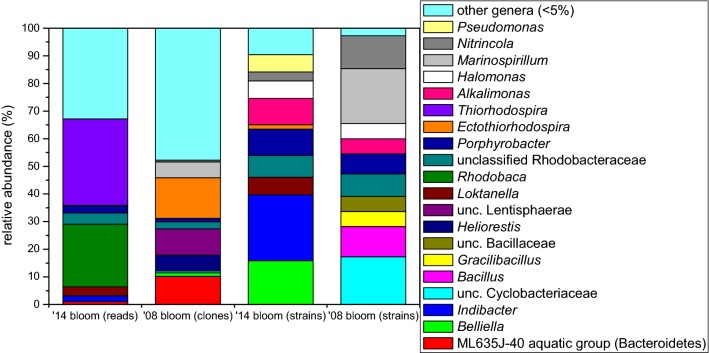
BCC of purple layer at genus level from Böddi-szék in 2008 (re-analysis of data published in Borsodi et al. 2013) and from an anonymous soda pan near Soltszentimre in 2014 based on different methods (cultivation, cloning and NGS). Only genera or equal ranks of uncultured bacteria with abundance above 5% are shown. Term ‘unc.’ stands for ‘unclassified’

From the three different solid media, 63 pure cultures were obtained, 51 with aerobic, 12 with anaerobic cultivation: 6 and 4 strains from medium ‘C’, 17 and 8 strains from medium ‘S’, 28 from ‘R’ agar plates, respectively. According to their 16S rDNA sequences, 37 strains belonged to phylum Proteobacteria (Alphaproteobacteria, 17 and Gammaproteobacteria, 20) and 26 strains to Bacteroidetes (Fig. 1). Isolates showed 89.0–100% sequence similarity values with type strains of validly published species (Table 2). No clear selectivity of the media was observed, while anaerobic cultivation resulted in similar bacteria as the aerobic one (e.g. *Halomonas*, *Porphyrobacter*, *Roseicitreum*), only few taxa were isolated exclusively under anaerobic conditions (represented by three strains showing the highest 16S rRNA gene similarities to *Erythromicrobium*, *Ectothiorhodospira* and *Adhaeribacter* type strains).

**Table 2:** Taxonomic affiliation of the bacterial strains isolated from the purple layer based on 16S rRNA gene sequence similarity

Phylum/Class	Order	Strain code	Closest species	16S rRNA gene similarity (%)	Presence of the *pufM* gene	Number of strains in sequence group
Proteobacteria Alphaproteobacteria	Sphingomonadales	S4B-2	*Erythromicrobium ramosum*	98.53	+	1
		S5B-2	*Porphyrobacter colymbi*/*donghaensis*	99.17	–	1
		R4-10P	*Porphyrobacter neustonensis*	99.05	only in C4B-1	4
	Rhodobacterales	S4-11	*Loktanella vestfoldensis*^*a*^	99.36	+	4
		R4-14	*Rhodobaca barguzinensis*	97.71	+	2
		S4-16	*Roseinatronobacter monicus*	97.71	+	1
		R3-13B	*Roseicitreum antarcticum*	99.58	+	2
		S5-6A	*Rhodobaculum claviforme*	99.87	–	1
	Rhizobiales	S4-27B	*Chelatococcus composti*	95.24	–	1
Gammaproteobacteria	Chromatiales	C3B-1	*Ectothiorhodospira shaposhnikovii*	100	+	1
	Oceanospirillales	R3-4	*Halomonas shengliensis*	98.97	–	1
	Oceanospirillales	S4-1	*Halomonas ventosae*	98.46	–	3
		R4-8^b^	*Nitrincola alkalisediminis*	98.56	–	2
	Pseudomonadales	R3-8	*Pseudomonas salegens*	98.87	–	1
		R4-10	*Pseudomonas salegens*	98.75	–	3
	Vibrionales	R4-7	*Vibrio metschnikovii*	99.70	–	3
	Alteromonadales	R4-13	*Alkalimonas amylolytica*	99.90	–	6
Bacteroidetes Cytophagia	Cytophagales	S4-3	*Belliella pelovolcani*	99.80	–	5
		R3-1	*Belliella aquatica*	97.28	–	5
	Cytophagales	R3-9	*Mongoliibacter ruber*	98.29	–	15
	Cytophagales	S4B-3	*Adhaeribacter aerophilus*	88.82	–	1

^a^*Loktanella vestfoldensis* was recently reclassified as *Yoonia vestfoldensis* by Wirth and Whitman (2018)

^b^This phylotype was recently described as *N. schmidtii* (Borsodi et al. 2017)

Vast majority of alphaproteobacterial strains were red colored (e.g. members of genera *Erythromicrobium*, *Porphyrobacter*, *Loktanella* and *Rhodobaculum*), four of them produced Bchl (Supplementary Figure S4). One of these strains, *Roseinatronobacter* sp. S4-16 stopped expressing its pigment(s) during the cultivation process. An anaerobic gammaproteobacterial strain showed 100% sequence similarity to an alkalophilic purple sulfur bacterium, *Ectothiorhodospira shaposhnikovii*. Other strains from this class were typical heterotrophs, with high similarities to halophilic or alkalophilic representatives of genera *Alkalimonas*, *Halomonas*, *Nitrincola*, *Pseudomonas* and *Vibrio*. All Bacteroidetes strains belonged to order Cytophagales, and were pink or bright red colored. Five strains were closely (99.8%) related to the type strain of *Belliella pelovolcani*, while another five strains probably represent a new species within this genus (~ 97% similarity to type strains). Fifteen strains were affiliated to *Mongoliibacter*, while strain S4B-3 may represent a new family, as the shared sequence similarity values to type strains were less than 89%.

Strains were screened for the presence of the *pufM* gene, and amplicons were obtained in the case of 12 strains (19% of total strains). As in many cases a longer fragment was co-amplified with the deposited amplicon (< 150 nt length), we presume an upstream, secondary annealing position of the forward (pufM_uniFfresh) primer inside the *pufL* gene. Taxon identification (against the GenBank database) based on *pufM* gene sequences was consistent to the 16S rDNA-based results. The *pufM*-positive strains belonged to the three purple bacterial groups [purple sulfur bacteria (PSB), purple nonsulfur bacteria (PNSB) and aerobic anoxygenic phototrophs (AAP)]. With the exception of the *Porphyrobacter* strains (one out of five was positive), strains from the same genus were either positive or negative. All *pufM*-containing isolates and representatives of sequence groups from *pufM*-negative strains were investigated with epifluorescence microscopy to detect the presence of Bchl. Not all of the *pufM*-positive strains were found to synthesize Bchl pigments, but Bchl was not detected in any of the *pufM*-negative strains. Interestingly, one strain (*Roseinatronobacter* sp. S4-16) stopped producing Bchl pigments during the cultivation process.

### Results of cultivation-independent analyses

Overall, 3398 high-quality sequences were obtained with next-generation DNA sequencing, which were clustered to 162 bacterial OTUs. Two OTUs with 27 sequences were identified as eukaryotic plastid and belonged to the chlorophyte genus *Oocystis* (unfortunately no *O. submarina* 16S rRNA gene sequence is available in GenBank currently; Supplementary Figure S5).

**Fig. 2:**
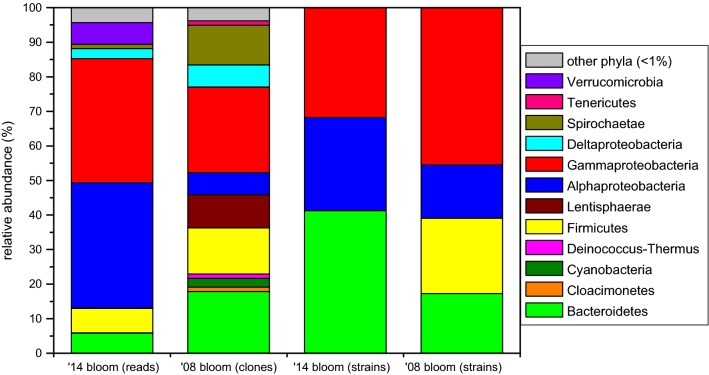
BCC of purple layer at phylum level from Böddi-szék in 2008 (re-analysis of data published in Borsodi et al. 2013) and from an anonymous soda pan near Soltszentimre in 2014 based on different methods (cultivation, cloning and NGS). Only phyla or classes (in case of Proteobacteria) with abundance above 1% are shown

Altogether 18 bacterial phyla were detected (Fig. 2). Proteobacteria was the most abundant phylum, dominated by Alpha- (36.2% of total bacterial reads) and Gammaproteobacteria (35.9%), while only low relative abundance of Deltaproteobacteria was detected (2.9%), and the contribution of Betaproteobacteria was negligible (< 0.1%). Other bacterial phyla represented only a minor fraction of the bacterial community: Firmicutes (7.1%), Verrucomicrobia (6.3%), Bacteroidetes (5.8%), etc. (Figure 2). Almost one-third of the sequences was affiliated with the PSB family Ectothiorhodospiraceae (Gammaproteobacteria) and the vast majority of these sequences belonged to a single OTU assigned to the genus *Thiorhodospira*, while other five OTUs containing two to four sequences clustered into a *Thioalkalivibrio*, two *Ectothiorhodospira* and two unclassified groups (Fig. 1). Another third of the reads was affiliated with PNSB and AAP groups of the Rhodobacteraceae family (Alphaproteobacteria), dominated by a single OTU belonging to genus *Rhodobaca*, while a *Loktanella* and a *Porphyrobacter* OTU turned out to be also significant (~ 3% both). Other notable groups (with 1–4% relative abundance) were: two unclassified OTUs from each Firmicutes and Alphaproteobacteria; two verrucomicrobial OTUs (from *Haloferula* and a closely related unclassified genus) and one OTU from genera *Desulfonatronum* (Deltaproteobacteria), *Indibacter* (Bacteroidetes) and group ML602 J-51 (Actinobacteria). In total, 11 OTUs showed higher relative abundance than 1%, together they contributed 75.7% of the bacterial community.

From the 162 bacterial OTUs, 10 were shared between cultivated strains and the NGS library, which included 43 strains (68% of isolates) and 1221 sequences (36% of total reads). 20 strains forming seven OTUs were not detected by NGS.

Based on the result of qPCR, the 16S rRNA gene copy number of Archaea was below the detection limit (40 PCR cycles).

## Discussion

### Features of the dual bloom

At the time of sampling, this small pan was close to desiccation with a maximum depth of 5 cm. The low water level resulted in high conductivity, though the salinity value still fell into the hyposaline category according to Hammer’s (1986) classification system. Salinity is thought to have a major influence on microbial community composition (Wu et al. 2006; Sorokin et al. 2014), exceeding the effect of temperature and pH (Lozupone and Knight 2007). The high levels of TOC and inorganic nutrients (Table 1) suggest no bottom-up limitation, and are originated from the droppings of livestock (buffalo) or migrating aquatic birds (Boros et al. 2008). Therefore, algal blooms occur frequently in shallow soda pans (Somogyi et al. 2009), but the observed dual bloom (simultaneous presence of algae and purple bacteria) is uncommon (Borsodi et al. 2013).

The green layer observed on the surface was virtually a monoculture of the unicellular green alga *Oocystis submarina*. In general, pico-sized algae dominate the phytoplankton, and *O. submarina* is found to be rare in the soda pans of the Carpathian Basin (Somogyi et al. 2009, 2017; Pálffy et al. 2014), though it bloomed in a neighboring soda pan in 2008 with a purple layer formed beneath (Borsodi et al. 2013). Phytoplankton blooms undergo succession stages including the exudate profiles of the cells. At the developmental phase, low-molecular weight (LMW) compounds (including amino acids with high nitrogen content) dominate, while during the aging, algae release mainly high-molecular weight (HMW) macromolecules (such as polysaccharides, lipids and proteins) (Buchan et al. 2014).

As postulated by Tank et al. (2011), calm, sunny and warm conditions favor PSB blooms. The dense layer of algae hinders mixing and makes favorable light conditions for phototrophic purple bacteria in the deeper layer (Vörös et al. 1998; Stomp et al. 2007). Additionally, as heterotrophs consume all the oxygen, the chemocline rose from the sediment just below the green layer, turning the bottom of the pan anaerobic, being advantageous to the growth of (generally) anaerobic PSB from the Ectothiorhodospiraceae family (Imhoff 2006). Moreover, the proximity of sediment and the activity of sulfate-reducing bacteria provide sulfide for sulfur bacteria. As these habitats are turbid due to wind-induced mixing and groundwater upwelling (Boros et al. 2017) which provide competitive advantage to picoalgae (Somogyi et al. 2017), we assume that besides calm weather, other factors (e.g. other meteorological and hydromorphological conditions, geographical position, selective zooplankton grazing; Eiler et al. 2003; Horváth et al. 2014) had contributed to the development of the *Oocystis* bloom. Contrary to many saline lakes (e.g. Lake Shira or Lake Shunet; Rogozin et al. 2005), the presence of the purple layer is not permanent, but only an occasional event in the studied region.

### Purple bacteria in the purple layer

In the purple layer, the observed motile vibrios had similar cellular characteristics as the family Ectothiorhodospiraceae (Imhoff 2006). The purple layer was dominated by two OTUs, a PSB belonging to genus *Thiorhodospira* and a PNSB from genus *Rhodobaca*. Although PSB prefer anaerobic or microaerobic conditions (Imhoff, 2006), these bacteria have been reported previously from the proximity of algal blooms or cyanobacterial mats (Kompantseva et al. 2009; Borsodi et al. 2013). Because PSB are considered to have the major role in purple layer formation (Ollivier et al. 1994), we suppose that in this case, members of *Thiorhodospira* were the main bloom-formers, while other detected red-pigmented taxa (*Rhodobaca*, *Porphyrobacter*, *Indibacter*) found favorable milieu created by the algae or the PSB.

Members of the Ectothiorhodospiraceae family are well known from alkaline aquatic environments (Sorokin et al. 2004; Imhoff, 2006; Vavourakis et al. 2016); additionally, Sorokin et al. (2004) showed moderate halotolerance of isolated *Thiorhodospira* species. Interestingly, in an earlier blooming event in 2008 in a nearby soda pan (Böddi-szék; Borsodi et al. 2013, Fig. 1), no *Thiorhodospira* sequences were found, but *Ectothiorhodospira* was the major PSB component in the purple layer (with 14.6% relative abundance). However, regarding the bloom observed in 2014, one of the isolates showed 100% sequence similarity with the type strain of *Ectothiorhodospira shaposhnikovii*, but based on NGS this was a negligible PSB group in this case.

It should be noted that the second most abundant OTU based on NGS (‘Rhodobaca’ in Fig. 1) harbored two valid genera, *Rhodobaca* and *Roseinatronobacter*, as type strains belonging to these genera share high (98.3–99.0%) 16S rRNA gene sequence similarity values. Another problem of proper taxon assignment was observed in the case of ‘unclassified Rhodobacteraceae’ (Fig. 1), since bootstrap values of *Rhodobaca* and related strains did not reach the threshold required for genus-level identification. Order Rhobobacterales [including both PNSBs (e.g. *Rhodobaca*) and AAPs (e.g. *Roseinatronobacter* and *Loktanella*)] occur frequently in soda lakes (Vavourakis et al. 2016; Szabó et al. 2017). This is a metabolically diverse group with the ability to utilize a wide range of substrates (Moran et al. 2007). Several members favor LMW organic matter, including by-products of algae, and the association of these bacteria with phytoplankton was supported by many studies (e.g. Moran et al. 2007; Buchan et al. 2014). However, Sarmento et al. (2016) studying marine microbial communities found that not the quality but the quantity of organic matter has the major effect on Alphaproteobacteria.

Most of our isolates from this group (in total 12 bacterial strains) contained the *pufM* gene, which encodes one of the core proteins of the photosynthetic reaction center. The expression of the photosynthetic reaction center genes is considered to be highly dependent on environmental conditions (Jeanthon et al. 2011), which is a possible explanation regarding the lack of Bchl pigments in strains containing the *pufM* gene besides having an incomplete photosynthetic gene cluster.

### Other bacteria in the purple layer

Several isolated strains represented the major contributors of the bacterial community (*Rhodobaca*, *Loktanella*, *Porphyrobacter* and *Indibacter*), while others represented potentially new taxa (one of these has been described as a new species recently, *Nitrincola schmidtii*; Borsodi et al. 2017). This could be the result of the high nutrient and organic matter content of the sample and the application of newly designed media. The complete absence of certain isolated heterotrophic genera (*Alkalimonas*, *Halomonas*, *Nitrincola*, *Pseudomonas* and *Vibrio*) from the NGS amplicon library can be explained by their relatively low abundance and easy cultivability. On the other hand, a few highly similar sequences to these strains were obtained from other soda lakes in this region (Szabó et al. 2017), and these genera were among the first cultivated bacteria from soda lakes (Grant and Sorokin 2011).

In our study, relatively few groups dominated the bacterial community (two OTUs from genera *Thiorhodospira* and *Rhodobaca* proportionated 55% of the obtained NGS reads, representatives of Gammaproteobacteria and Alphaproteobacteria, respectively). Our earlier investigations on nearby soda pans (Szabó et al. 2017; Szabó A., Korponai K., and Felföldi T., unpublished results) showed that Actinobacteria is the most abundant phylum along with Bacteriodetes and class Alphaproteobacteria. All these results support that dynamic changes of environmental parameters at these sites (Kirschner et al. 2002; Felföldi et al. 2009; Somogyi et al. 2009; Boros et al. 2017) induce drastic changes in the composition of planktonic prokaryotes (including occasional blooms).

The different oxygen tolerance of genera detected in the purple layer (*Loktanella* and *Indibacter* are obligate aerobic, *Thiorhodospira* and *Desulfonatronum* are obligate anaerobes, while *Rhodobaca* can switch from one to another using different electron acceptors; Koblížek 2015) suggests that an aerobic–anaerobic gradient was present within this 2-cm-thick layer. *Desulfonatronum* is the most widely reported sulfate-reducing bacterial genus from soda lakes (Sorokin et al. 2011), which supports the electron donor (sulfide) to PSB and PSNB.

Bacteroidetes species have a major role in organic matter biodegradation with the preference of complex, recalcitrant (HMW) molecules (Cottrell and Kirchman 2000; Pérez and Sommaruga 2006). While most of the detected Bacteroidetes bacteria are considered to be obligate aerobes (e.g. *Indibacter*, *Belliella*, *Aquiflexum*), members of the probably anaerobic ML635 J-40 aquatic group (eight OTUs with 9.8% relative abundance altogether) (Humayoun et al. 2003) was also identified. We suppose that aerobic Bacteroidetes bacteria were associated with algae; their presence in the deeper zone can be explained by gravitational sinking from the green layer.

The dominant Actinobacteria OTU (ML602 J-51) was found to be an infrequent member of the otherwise prevalent phylum in the Central European soda pans (Szabó et al. 2017, Szabó A., Korponai K. and Felföldi T., unpublished results). Phylum Verrucomicrobia (e.g. *Haloferula*) have been reportedly found to be associated with blooming events (Eiler and Bertilsson 2004), and members of this phylum could contribute with 2–10% to soda pan bacterial communities (Szabó et al. 2017; this study).

Comparing to other soda lake studies (Grant 2006; Antony et al. 2013; Borsodi et al. 2013), the complete absence of *Bacillus* and related species in our isolates and NGS reads is striking, though the applied method is suitable to their identification (Kalwasińska et al. 2017). Most of the detected Firmicutes sequences (66%) fell into a yet uncultured genus of order Clostridiales, while the phylum itself seems to be negligible (7%), though this value exceeds our previous findings (0.2–1.5%) (Szabó et al. 2017).

The negligible proportion of Archaea can be a result of the relatively low salinity (between 3.8 and 24.6 g/L; Table 1) of the pan (Oren 1994).

### Dual blooms in the region

Comparing the revealed BCC in this anonymous soda pan with a previous *Oocystis*-associated purple bacterial bloom in a neighboring soda lake (Böddi-szék), significant differences were found (Figs. 1 and 2). While both communities were dominated by PSB, different genera were identified. Other, functionally similar groups also showed habitat separation: *Belliella* was present in the previous, while in the former blooming event it was replaced by its close relative *Indibacter*. *Rhodobaca* was absent and other alphaproteobacteria composed only a minor fraction in Böddi-szék, where a yet uncultured Bacteroidia group (ML635 J-40 aquatic group) showed higher relative abundance. Some important groups in the BCC of Böddi-szék, such as heliobacteria, Lentisphaerae, and heterotrophic gammaproteobacteria were absent in the community of the anonymous soda pan. On the contrary, *Loktanella* was present only in the latter. The re-analysis of the originally published data (Borsodi et al. 2013) enabled us to compare the two datasets generated through different methods. It is also noteworthy, that in the re-analyzed dataset many taxa were assigned to different genera than originally, which highlights the need to avoid outdated taxonomy, and the fact, that the field of soda lake research is still thriving.

It seems that temperature (Kirschner et al. 2002) could be the key selection factor for PSB, not only in the Chromaticeae (Tank et al. 2011) but in the Ectothiorhodospiraceae family as well, since water temperature was higher in the case of Böddi-szék (33 °C; Borsodi et al. 2013) than in the case of the anonymous soda pan (23 °C) bloom, and the growth temperature optimum of *Ectothiorhodospira* strains is usually above, while for *Thiorhodospira* is below 30 °C (Oren 2014). Another explanation could be the different ionic compositions of the two lakes, since Böddi-szék is a ‘soda-saline’ type (Na^+^, Cl^−^ > HCO_3_^−^), while the anonymous pan belongs to the ‘soda’ type (Na^+^, HCO_3_^−^ > Cl^−^) according to the classification of Boros and Kolpakova (2018).

## Summary

A dual bloom of green algae and purple bacteria in a Central European soda pan was characterized in this study. While algal blooms are common in these waters, the presence of a purple layer is only an occasional event in the studied region. The high level of oxygen produced by the algae was consumed by heterotrophic bacteria in a few centimeters, and a sulfuretum was developed, where sulfate reducers created hydrogen sulfide, which was oxidized to sulfate by PSBs, PNSBs and AAPs. Probably the warm, calm and sunny conditions enabled both the *Oocystis* bloom, and the development of a sulfuretum in the water as the chemocline rose into the water column from the sediment–water boundary. These special conditions resulted in an uncommon microbial community, dominated by PSB (*Thiorhodospira*) and PNSB (*Rhodobaca*) that oxidize sulfide under anaerobic conditions. The purple layer development was probably initiated by the sulfide genesis of sulfate-reducing bacteria (*Desulfonatronum*).

It can be concluded that such purple layer formation in a shallow lake is a result of the combination of several biotic and abiotic factors (e.g. favorable light climate, ionic composition, calm weather, sediment proximity).

## Electronic supplementary material

Below is the link to the electronic supplementary material.

Supplementary material 1 (PDF 542 kb)

## Abbreviations

AAP: Aerobic anoxygenic phototrophs
BCC: Bacterial community composition
Bchl: Bacteriochlorophyll(s)
CFU: Colony forming units
Chl *a*: Chlorophyll *a*
HMW: High molecular weight
LMW: Low molecular weight
NGS: Next-generation DNA sequencing
NTU: Nephelometric turbidity unit
OTU: Operational taxonomic unit
PNSB: Purple non-sulfur bacteria
PSB: Purple sulfur bacteria
SRP: Soluble reactive phosphorus
TN: Total nitrogen
TOC: Total organic carbon
