# Supplementary material for: Dual bloom of green algae and purple bacteria in an extremely shallow soda pan
Source: Extremophiles. 2019 May 13;23(4):467–77. doi: 10.1007/s00792-019-01098-4 (PMC6557878; doi:10.1007/s00792-019-01098-4)
Supplement: Supplementary file 1 — Supplementary material 1 (PDF 542 kb) [file 792_2019_1098_MOESM1_ESM.pdf]

ELECTRONIC SUPPLEMENTARY MATERIAL

for

Kristóf Korponai, Attila Szabó, Boglárka Somogyi, Emil Boros, Andrea K. Borsodi, Laura Jurecska, Lajos Vörös, Tamás Felföldi

Dual bloom of green algae and purple bacteria in an extremely shallow soda pan

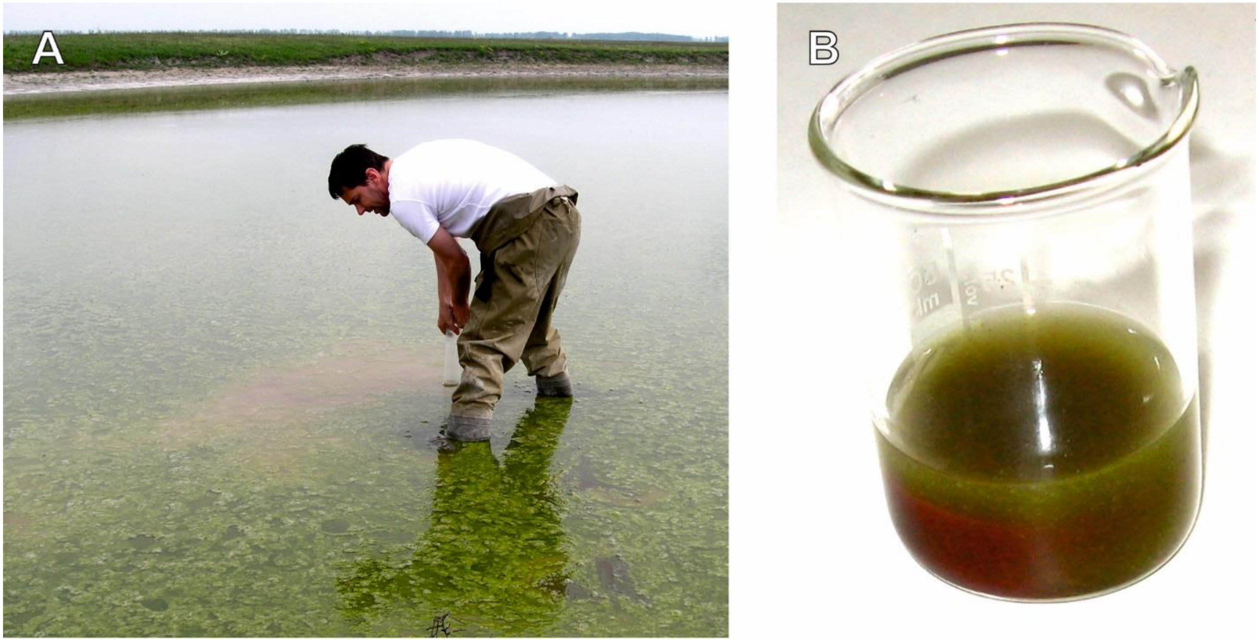

Supplementary Fig. S1. Macroscopic images of a dual bloom occurred in an anonymous soda pan near Soltszentimre in April 2014. A – sample collection, B – negative phototaxis (high light intensity from the right side) and negative aerotaxis of purple-colored cells after mixing the two layers in a beaker

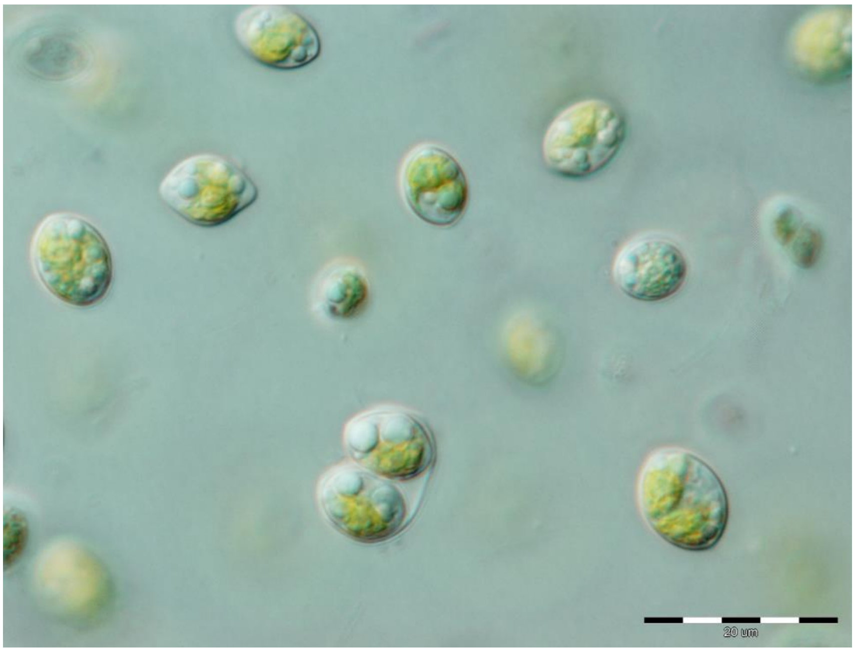

Supplementary Fig. S2. Micrograph (differential interference contrast, DIC) of the green layer (cells of *Oocystis submarina*)

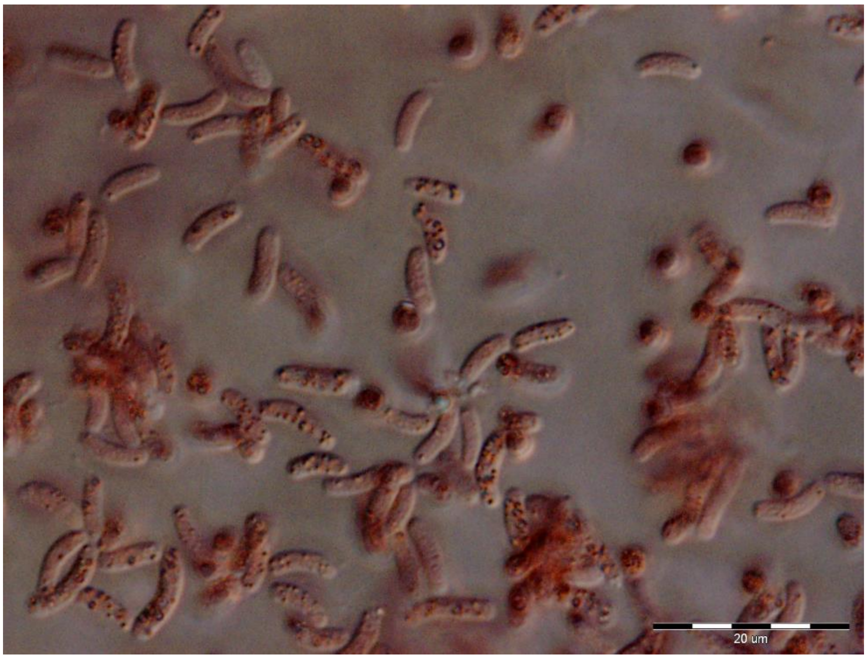

Supplementary Fig. S3. Micrograph (DIC) of the purple layer

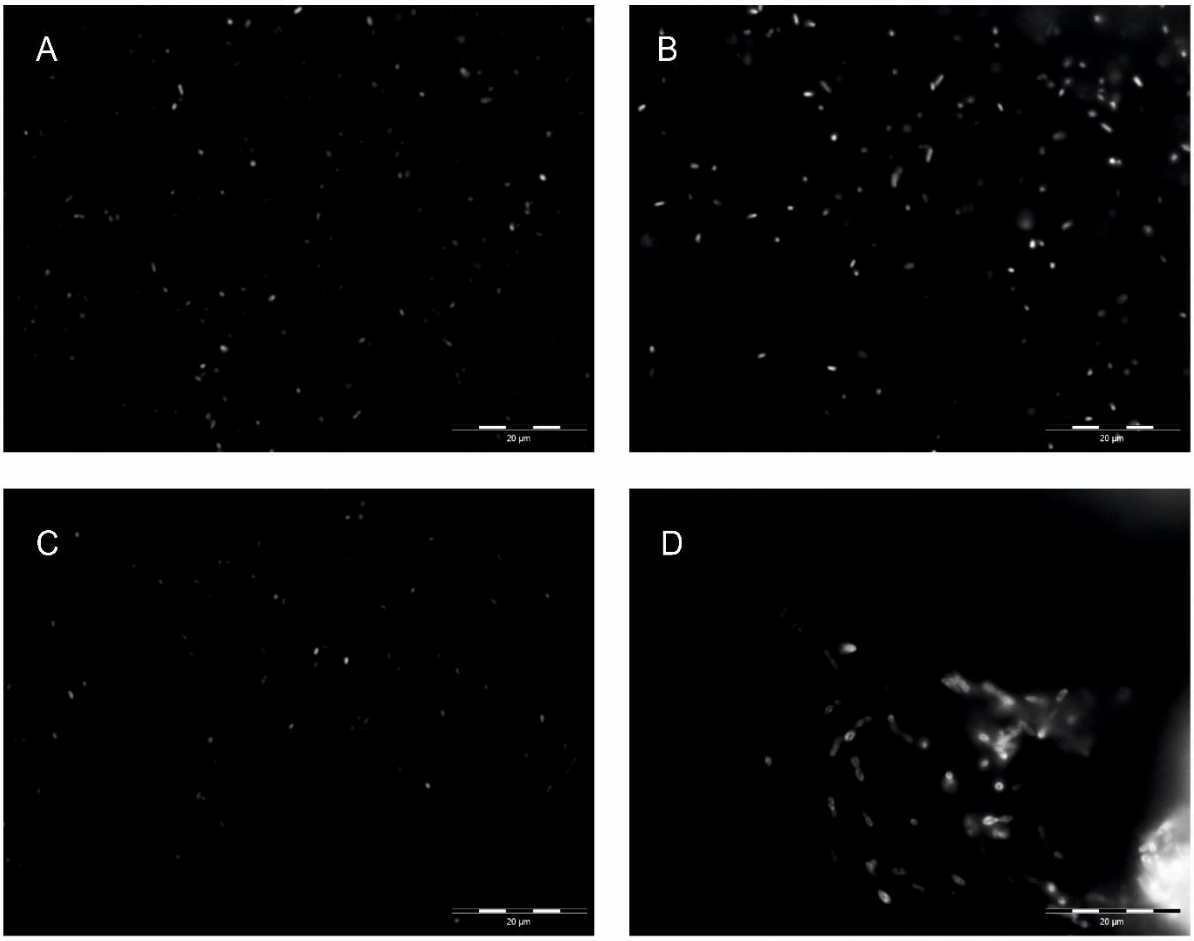

Supplementary Fig. S4. Micrographs of bacterial strains using infrared camera (excitation: 350-550 nm, emission: >780 nm). A - *Loktanella vestfoldensis* C4-6; B - *Loktanella vestfoldensis* S4-14B; C - *Roseinatronobacter* sp. S4-16; D - *Rhodobaculum claviforme* S5-6A.

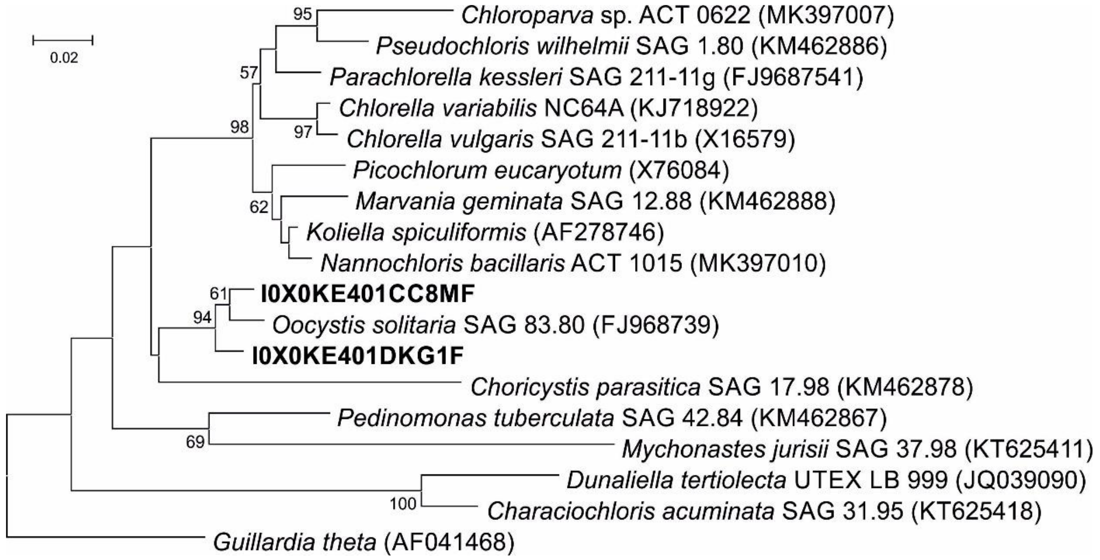

Supplementary Fig. S5. Neighbor-joining phylogenetic tree of chloroplast genotypes obtained from the purple layer of a dual bloom in an anonymous soda pan near Soltszentimre based on NGS of the 16S rRNA gene. Tree was constructed using the Kimura 2- parameter nucleotide substitution model and is based on 431 nucleotide positions.
